# An evaluation of multidrug-resistant *Escherichia coli* isolates in urinary tract infections from Aguascalientes, Mexico: cross-sectional study

**DOI:** 10.1186/s12941-018-0286-5

**Published:** 2018-07-24

**Authors:** Flor Y. Ramírez-Castillo, Adriana C. Moreno-Flores, Francisco J. Avelar-González, Francisco Márquez-Díaz, Josée Harel, Alma L. Guerrero-Barrera

**Affiliations:** 1 Laboratorio de Biología Celular y Tisular, Departamento de Morfología, Universidad Autónoma de Aguascalientes, Av. Universidad 940, Col. Cd. Universitaria, 20131 Aguascalientes, Mexico; 2 Laboratorio de Ciencias Ambientales, Departamento de Fisiología y Farmacología, Universidad Autónoma de Aguascalientes, Av. Universidad 940, Col. Cd. Universitaria, 20131 Aguascalientes, Mexico; 3 Departamento de Infectología, Centenario Hospital Miguel Hidalgo, Galeana Sur 495, Obraje, 20000 Aguascalientes, Mexico; 4 Département de pathologie et microbiologie, Centre de Recherche en Infectologíe Porcine et Aviaire, Faculté de Médecine Vétérinaire, Université de Montréal, 3200, rue Sicott, Saint-Hyacinthe, Montreal, QC J2S 2M2 Canada

**Keywords:** Urinary tract infection, Multidrug-resistant, Fluoroquinolone-resistant, Plasmid-mediated quinolone resistance (PMQR), CTX-M

## Abstract

**Background:**

Uropathogenic *Escherichia coli* (UPEC) are one of the main bacteria causing urinary tract infections (UTIs). The rates of UPEC with high resistance towards antibiotics and multidrug-resistant bacteria have increased dramatically in recent years and could difficult the treatment.

**Methods:**

The aim of the study was to determine multidrug-resistant bacteria, antibiotic resistance profile, virulence traits, and genetic background of 110 *E. coli* isolated from community (79 isolates) and hospital-acquired (31 isolates) urinary tract infections. The plasmid-mediated quinolone resistance genes presence was also investigated. A subset of 18 isolates with a quinolone-resistance phenotype was examined for common virulence genes encoded in diarrheagenic and extra-intestinal pathogenic *E. coli* by a specific *E. coli* microarray.

**Results:**

Female children were the group most affected by UTIs, which were mainly community-acquired. Resistance to trimethoprim–sulfamethoxazole, ampicillin, and ampicillin–sulbactam was most prevalent. A frequent occurrence of resistance toward ciprofloxacin (47.3%), levofloxacin (43.6%) and cephalosporins (27.6%) was observed. In addition, 63% of the strains were multidrug-resistant (MDR). Almost all the fluoroquinolone (FQ)-resistant strains showed MDR-phenotype. Isolates from male patients were associated to FQ-resistant and MDR-phenotype. Moreover, hospital-acquired infections were correlated to third generation cephalosporin and nitrofurantoin resistance and the presence of *kpsMTII* gene. Overall, *fimH* (71.8%) and *fyuA* (68.2%), had the highest prevalence as virulence genes among isolates. However, the profile of virulence genes displayed a great diversity, which included the presence of genes related to diarrheagenic *E. coli*. Out of 110 isolates, 25 isolates (22.7%) were positive to *qnrA*, 23 (20.9%) to *qnrB*, 7 (6.4%) to *qnrS1*, 7 (6.4%) to *aac(6′)lb*-*cr*, 5 (4.5%) to *qnrD*, and 1 (0.9%) to *qnrC* genes. A total of 12.7% of the isolates harbored *bla*_CTX-M_ genes, with *bla*_CTX-M-15_ being the most prevalent.

**Conclusions:**

Urinary tract infection due to *E. coli* may be difficult to treat empirically due to high resistance to commonly used antibiotics. Continuous surveillance of multidrug resistant organisms and patterns of drug resistance are needed in order to prevent treatment failure and reduce selective pressure. These findings may help choosing more suitable treatments of UTI patients in this region of Mexico.

## Background

*Escherichia coli* is an important cause of extra-intestinal infections, enteric disease, and systemic infections in humans and animals. Uropathogenic *Escherichia coli* (UPEC), one of the members of the extra-intestinal pathogenic *E. coli* (ExPEC) is a predominant pathogen causing urinary tract infections (UTIs) [1]. UPEC is one of the main causes of community (80–90%) and nosocomial-acquired UTIs (30–50%) [2]. These strains harbor a variety of virulence factors that allow them to establish an infection, including adhesins, toxins, host defense avoidance mechanisms and multiple iron acquisition systems [3].

UTIs are one of the most common bacterial infections worldwide. It has been estimated that 150 million UTIs occur per year worldwide [4]. ExPEC strains are responsible for an estimated 40,000 deaths and at least $2.6 billion of expenses in healthcare treatment in the United States alone [5]. In Mexico, 3’648,784 cases were reported on 2015 [6]. Although these infections are treatable, the increasingly accelerated rates or multi-drug resistant organisms (MDRO) lead to complication, treatment failure and increased rates of mortality and morbidity [7].

The enhanced prevalence and global spread of extended-spectrum beta-lactamase (ESBL) genes [8] such as CTX-M enzymes that are associated to multidrug-resistant (MDR) phenotypes and fluoroquinolones resistance, has become a major concern [9, 10]. Quinolones are broad-spectrum antibiotics and very important in the treatment of a wide range of diseases, especially urinary tract infections. Quinolone resistance is mainly caused by chromosomal mutations in the quinolone resistance-determining region (QRDR) of gyrase (*gyrA* and *gyrB*) and topoisomerase (*parC* and *parE*) genes, but also is caused by plasmid-mediated quinolone resistance (PMQR) which include: the pentapeptide repeat family Qnr proteins (QnrA, QnrB, QnrS, QnrC and QnrD) [11], the variant of the aminoglycoside-acetyltransferase modifying enzyme, AAC(6′)-lb-cr [12], and the efflux pumps QepA and OqxAB [13–15]. Although PMQR genes confer only low-level of resistance to quinolones [16] they could spread horizontally among *Enterobacteria* and facilitate the selection of resistant mutants as well as the selection of additional resistance mechanisms, enabling bacteria to become fully resistant [8, 17].

The emergence of MDR *E. coli* causing urinary tract infections with high virulence potential is alarming. In Mexico, a high rate of resistance against quinolones has been observed in environmental, diarrheagenic [18], and pediatric clinical isolates [19]. MDR phenotype of UPEC has also been shown [20, 21]. However, lack of sufficient data on virulence spectrum and MDR-UPEC isolates from community and hospital infections hinders the infections control and management efforts. The aim of this study was to determine the occurrence of MDR-phenotype, antibiotic resistance profile, virulence traits, and genetic background of *E*. *coli* isolates from community and hospital-acquired UTIs from Mexico.

## Methods

### *Escherichia coli* isolation

This study was conducted on a total of one hundred and ten urine cultures collected from patients suspected to have an UTI (urine samples contained bacterial counts to ≥ 10^5^ CFU/ml) and sought attention in the Centenario Hospital Miguel Hidalgo, in Aguascalientes, Mexico during the period June to November 2012. Patient’s ages ranged from neonatal to 91 years. Only one non-duplicated *E. coli* isolate per culture was considered. Patients from whom *E. coli* was isolated at least 48 h after admission were considered to have a hospital-acquired infection, all other infections were considered to be community-acquired [22]. To isolate *E. coli*, serial decimal dilutions of the sample were prepared in 0.85% NaCl, and were used to inoculate MacConkey agar plates which were incubated overnight at 37 °C. All the isolates were screened by PCR for the *uidA* gene as confirmation (Table 1). The *E. coli* isolates were stored at − 80 °C in Luria–Bertani broth and 20% (vol/vol) glycerol.

**Table 1:** Oligonucleotides used in this study

Oligonucleotide name	Target gene	Oligonucleotide 5′ ➔ 3′	Amplification product (bp)	References
*E. coli* marker				
uidA-forward	*uidA*	ATGTGCTGTGCCTGAACC	450	[60]
uidA-reverse		ATTGTTTGCCTCCCTGCTG		
Virulence genes for extra-intestinal pathogenic *E. coli*				
papC-forward	*papC*	GACGGCTGTACTGCAGGGTGTGGCG	350	[61]
papC-reverse		ATATCCTTTCTGCAGGGATGCAATA		
SfaSf	*sfaS*	GTGGATACGACGATTACTGTG	240	[62]
SfaSr		CCGCCAGCATTCCCTGTATTC		
Afaf	*afa/dra*	GGCAGAGGGCCGGCAACAGGC	592	[62]
Afar		CCCGTAACGCGCCAGCATCTC		
FyuAf	*fyuA*	TGATTAACCCCGCGACGGGAA	880	[62]
FyuAr		CGCAGTAGGCACGATGTTGTA		
yfcV-forward	*yfcV*	ACATGGAGACCACGTTCACC	292	[63]
yfcV-reverse		GTAATCTGGAATGTGGTCAGG		
Vat-forward	*vat*	TCAGGACACGTTCAGGCATTCAGT	1100	[63]
Vat-reverse		GGCCAGAACATTTGCTCCCTTGTT		
KpsMIIf	*kpsMT II*	GCGCATTTGCTGATACTGTTG	272	[62]
KpsMIIr		CATCCAGACGATAAGCATGAGCA		
Quinolone resistance genes				
gyrA11753	*gyrA*	GTATAACGCATTGCCGC	251	[64]
gyrA12004		TGCCAGATGTCCGAGAT		
EC-PAR-A	*parC*	CTGAATGCCAGCGCCAAATT	189	[65]
EC-PAR-B		GCGAACGATTTCGGATCGTC		
qnrA-forward	*qnrA*	TCAGCAAGAGGATTTCTCA	605	[66]
qnrA-reverse		GGCAGCACTATTACTCCCA		
qnrB-forward	*qnrB*	GATCGTGAAAGCCAGAAAGG	469	[12]
qnrB-reverse		ACGATGCCTGGTAGTTGTCC		
qnrS-forward	*qnrS*	ACGACATTCGTCAACTGCAA	417	[12]
qnrS-reverse		TAAATTGGCACCCTGTAGGC		
qnrC-forward	*qnrC*	GGGTTGTACATTTATTGAATC	447	[64]
qnrC-reverse		TCCACTTTACGAGGTTCT		
qnrD-forward	*qnrD*	CGAGATCAATTTACGGGGAATA	582	[27]
qnrD-reverse		AACAAGCTGAAGCGCCTG		
qepA-forward	*qepA*	CTGCAGGTACTGCGTCATG	403	[67]
qepA-reverse		CGTGTTGCTGGAGTTCTTC		
aac-forward	*acc*-*(6′)*-*lb*	TTGCGATGCTCTATGAGTGGCTA	482	[26]
aac-reverse		CTCGAATGCCTGGCGTGTTT		
Beta-lactamase resistant genes				
blaTEM-forward	*bla*_TEM_	GAGTATTCAACATTTTCGT	857	[30]
blaTEM-reverse		ACCAATGCTTAATCAGTGA		
blaSHV-forward	*bla*_SHV_	TCGCCTGTGTATTATCTCCC	768	[30]
blaSHV-reverse		CGCAGATAAATCACCACAATG		
blaOXA-1-forward	*bla*_OXA-1_	GCAGCGCCAGTGCATCAAC	198	[30]
blaOXA-1-reverse		CCGCATCAAATGCCATAAGTG		
blaOXA-7-forward	*bla*_OXA-7, -9_	AGTTCTCTGCCGAAGCC	591	[30]
blaOXA-7-reverse		TCTCAACCCAACCAACCC		
blaPSE-4-forward	*bla*_PSE-4_	CTGCTCGTATAGGTGTTTCC	705	[30]
blaPSE-4-reverse		TCGCATCATTTCGCTCTTC		
blaCTX-M-3-f	*bla*_CTX-M-3_	AATCACTGCGTCAGTTCAC	701	[30]
blaCTX-M-3-r		TTTATCCCCCACAACCCAG		
blaCTX-M-forward	*bla*_CTX-M_	AAGGCGTTTTGACAGACTATT	920	This study
blaCTX-M-reverse		GGTGACGATTTTAGCCGC		

### Antimicrobial susceptibility test

Susceptibility to antimicrobial agents was determined by the agar dilution method as recommended by the standards of Clinical Laboratory Standards Institute [23]. The following antimicrobial agents were tested: ampicillin, ampicillin–sulbactam, amoxicillin–clavulanic acid, piperacillin–tazobactam, cefazolin, cefotaxime, ceftazidime, ceftriaxone, cefepime, amikacin, gentamicin, tobramycin, netilmicin, imipenem, ertapenem, trimethoprim–sulfamethoxazole, ciprofloxacin, levofloxacin and norfloxacin. *E. coli* ATCC 25922 was included in each assay as a negative control.

### Determination of phylo-groups

Phylo-grouping was determined by PCR as previously described by Clermont et al. [24]. *E. coli* strains H10407 (phylo-group A), E22 (phylo-group B1), CFT073 (phylo-group B2), ECOR 70 (phylo-group C), O42 (phylo-group D), EDL933 (phylo-group E) and ECOR 36 (phylo-group F), were taken as positive controls. Water was used as negative control. All strains controls were kindly provided by Laboratoire de référence pour *Escherichia coli*, EcL, Faculté de Médecine Vétérinaire, Université de Montréal.

### Detection of virulence factors

*Escherichia coli* isolates from urinary tract infections were screened for the presence of commonly detected UPEC virulence genes including: *fyuA*, *fimH*, *sfaS*, *afa/dra*, *papC*, *agn43, yfcV*, *vat*, *hlyA*, *cnf1*and *kpsMTII*. Primers and controls used in this study are listed in Tables 1 and 2.

**Table 2:** PCR control strains used in this study

Control strain	Positive gene (s)
J53pMG252	*qnrA*
J53pMG298	*qnrB*
J53pMG306	*qnrS*
*Salmonella* SA20042859	*aac(6′)*-*Ib*
CFT073	*uidA, papC, fyuA, chuA, kpsMTII, yfcV*
J96	*sfaS*
UTI89	*vat*
R455	*bla*_OXA-1_
R6 K	*bla*_TEM_
HB101	*bla*_SHV_
pMG202	*bla*_OXA-7, -9_
pMON711	*bla*_PSE-4_
CCRI-2167	*bla*_CTX-M_

### Characterization of resistance genes and mutations

Screening for plasmid mediated quinolone resistance (PMQR) genes, including *qnrA*, *qnrB*, *qnrS*, *qnrC, qnrD*, *qepA*, and *acc(6′)*-*lb* genes was performed as previously described [13, 25–28]. The *qnrC*, *qnrD* and *aac (6′)*-*Ib cr* variants were identified sequencing the PCR products. The quinolone resistance determining regions (QRDR) of *gyrA* and *parC* genes were amplified and sequenced on both strands and predicted peptide sequences were compared to the corresponding gene from the MG1655 genome as described by Namboodiri et al. [29]. Beta-lactams genes detection including *bla*_TEM_, *bla*_SHV_, *bla*_OXA_, *bla*_PSE_ and *bla*_CTX-M_ were carried out by PCR amplification using specific primers [30]. Amplicons from all of the *bla*_CTX-M_ positive isolates were sequenced. The nucleotide sequences obtained were aligned and further analyzed by comparison to sequences from a catalogue of beta-lactamases (http://www.lahey.org/studies/).

### DNA microarray analysis

Microarray hybridizations were performed using *E. coli* maxivirulence version 3.1 microarray as previously described [31]. It allows the detection of 348 virulence genes and 98 antibiotic resistance genes and variants. DNA extraction and hybridizations were performed as described previously [32]. Each isolate was assigned to a specific *E. coli* pathotype according to its virulence gene profile and based on the classification published in previous papers [31, 33].

### Statistical analysis

Categorical variables were compared using Pearson’s Chi square or Fisher’s exact test as appropriate. Continuous variables were compared using Mann–Whitney U test (STATISTICA V. 10, StatSoft, United States). p values less than 0.05 were considered statistically significant.

## Results

### Patient demographics

During the period of study, females (86 samples, 78.2%) were the most affected group of patients (*p* < 0.05) as compared to males (24 samples, 21.8%). Most of the UTIs were detected in community obtained samples (79 samples, 71.8%). Thirty-one samples were recovered from hospital-acquired infections (28.2%). *E. coli* isolated from female patients were correlated with community-acquired UTIs (*p* = 0.0297). No differences among the prevalence of *E. coli* isolated from male patients among community- and hospital-acquired infections were found (54.2% vs 45.8%, respectively). Maximum number of cases was found in female children in the age group of 2–10 years (34 samples, 39%, *p* = 0.0372, Table 3).

**Table 3:** Patients with urinary tract infections, n (%)

Age group	All patients, *n *= 110 (%)	Male, *n* = 24 (%)	Female, *n* = 86 (%)	*p	Hospital-acquired, *n* = 31 (%)	Community-acquired, *n* = 79 (%)	*p
0–1	17 (15.5)	6 (25.0)	11 (12.8)	0.1434	5 (16.1)	12 (15.2)	0.9024
2–10	38 (34.5)	4 (16.7)	34 (39.5)	*0.0372*	9 (29.0)	29 (36.7)	0.4462
11–20	12 (10.9)	1 (4.2)	11 (12.8)	0.2308	2 (6.5)	10 (12.7)	0.3475
21–30	7 (6.4)	2 (8.3)	5 (5.8)	0.6548	1 (3.2)	6 (7.6)	0.3983
31–40	6 (5.5)	1 (4.2)	5 (5.8)	0.7533	1 (3.2)	5 (6.3)	0.5191
41–50	5 (4.5)	0 (0.0)	5 (5.8)	0.2266	1 (3.2)	4 (5.1)	0.6772
51–60	6 (5.5)	2 (8.3)	4 (4.7)	0.4824	3 (9.7)	3 (3.8)	0.2218
61–70	7 (6.4)	3 (12.5)	4 (4.7)	0.1636	3 (9.7)	4 (5.1)	0.3724
> 71	12 (10.9)	5 (20.8)	7 (8.1)	0.0777	6 (19.4)	6 (7.6)	0.0751

* Italic number shown statistical significant values

### Antibiotic resistance and MDR profiles in *E. coli* isolates

High frequencies of resistance were observed toward trimethoprim–sulfamethoxazole (72.7%), ampicillin (70.9%), ampicillin–sulbactam (55.5%), piperacillin–tazobactam (55.5%), ciprofloxacin (47.3%), and levofloxacin (43.6%). From the twenty different antibiotic tested, all isolates were susceptible to carbapenems (ertapenem and imipenem, Table 4). Eighty-seven percent of the isolates were resistant to at least one antimicrobial agent, and 63.3% (70 isolates) were multidrug-resistant (MDR). MDR isolates were resistant to ampicillin (85.7%), trimethoprim–sulfamethoxazole (84.3%), ampicillin–sulbactam (77.1%), piperacillin–tazobactam (77.1%) and ciprofloxacin (70%, Table 4). A total of 94.2% (49/52 isolates) of FQ-resistant strains showed MDR-phenotype. A similar distribution of FQ-resistant isolates was found in the same at hospital as community-acquired infections, except for norfloxacin, which was higher among community-acquired infections (20.3% vs 6.5%, Table 4). Distribution of ciprofloxacin and levofloxacin resistance was significantly higher among male than female gender (70.8, 66.7%, *p *= 0.0089, 0.0314 vs 40.7, 37.2%, respectively, Table 4).

**Table 4:** Antibiotic and multidrug resistance of the UPEC’s isolates, n (%)

Antibiotics	Total isolates, *n* = 110 (%)	MDR, *n* = 70 (%)	Male, *n* = 24 (%)	Female, *n* = 86 (%)	*p	Hospital-acquired, *n* = 31 (%)	Community-acquired, *n* = 79 (%)	*p
Amikacin	11 (10.0)	11 (15.9)	4 (16.7)	7 (8.1)		1 (3.2)	10 (12.7)	
Gentamicin	31 (28.2)	29 (41.4)	11 (45.8)	20 (23.3)	0.0297	10 (32.3)	21 (26.6)	
Tobramycin	21 (19.1)	21 (30.0)	10 (41.7)	11 (12.8)	0.0015	9 (29.0)	12 (15.2)	
Netilmicin	6 (5.5)	5 (7.1)	2 (8.3)	4 (4.7)		2 (6.5)	4 (5.1)	
Ampicillin	78 (70.9)	60 (85.7)	20 (83.3)	58 (67.4)		24 (77.4)	54 (68.4)	
Ampicillin–sulbactam	61 (55.5)	54 (77.1)	15 (62.5)	46 (53.5)		18 (58.1)	43 (54.4)	
Amoxicillin–clavulanic acid	26 (23.6)	23 (32.9)	5 (20.8)	21 (24.4)		6 (19.4)	20 (25.3)	
Piperacillin–tazobactam	61 (55.5)	54 (77.1)	15 (62.5)	46 (53.5)		18 (58.1)	43 (54.4)	
Cefazolin	46 (41.8)	45 (64.3)	15 (62.5)	31 (36.0)	0.0202	16 (51.6)	30 (38.0)	
Cefotaxime	20 (18.2)	20 (28.6)	5 (20.8)	15 (17.4)		6 (19.4)	14 (17.7)	
Ceftazidime	27 (24.5)	27 (38.6)	13 (54.2)	14 (16.3)	0.0001	12 (38.7)	15 (19.0)	0.0306
Ceftriaxone	30 (27.3)	30 (42.9)	14 (58.3)	16 (18.6)	0.0001	13 (41.9)	17 (21.5)	0.0305
Cefepime	29 (26.4)	29 (41.4)	14 (58.3)	15 (17.4)	0.0001	12 (38.7)	17 (21.5)	
Trimethoprim–sulfamethoxazole	80 (72.7)	59 (84.3)	18 (75.0)	62 (72.1)		24 (77.4)	56 (70.9)	
Ciprofloxacin	52 (47.3)	49 (70.0)	17 (70.8)	35 (40.7)	0.0089	15 (48.4)	37 (46.8)	
Levofloxacin	48 (43.6)	45 (64.3)	16 (66.7)	32 (37.2)	0.0314	13 (41.9)	35 (44.3)	
Norfloxacin	18 (16.4)	18 (25.7)	3 (12.5)	15 (17.4)		2 (6.5)	16 (20.3)	
Nitrofurantoin	14 (12.7)	12 (17.1)	5 (20.8)	9 (10.5)		4 (12.9)	10 (12.7)	0.0002
Ertapenem	0 (0.0)	0 (0.0)	0 (0.0)	0 (0.0)		0 (0.0)	0 (0.0)	
Imipenem	0 (0.0)	0 (0.0)	0 (0.0)	0 (0.0)		0 (0.0)	0 (0.0)	

* Only statistical significant values are shown

Co-resistance phenotype between cephalosporins and beta-lactams towards fluoroquinolones was frequently observed [(amoxicillin–clavulanic acid (*p* = 0.0004), cefazolin (*p* = 0.0299), cefotaxime (*p* = 0.0001), ceftriaxone (*p* = 0.0002) and, cefepime (*p* = 0.0015)]. Trimethoprim–sulfamethoxazole and cephalosporins [cefotaxime (*p* = 0.0232) and ceftazidime (*p* = 0.0001)] also showed a co-resistance.

Overall, hospital-acquired isolates were slightly more resistant than community-acquired isolates (33.1% vs 28.7%, respectively). Both hospital and community-acquired isolates showed higher resistance towards ampicillin (77.4% vs 68.4%) and trimethoprim–sulfamethoxazole (77.4% vs 70.9%). UPEC isolates from hospital-acquired infections were associated to ceftazidime (*p* = 0.0306), ceftriaxone (*p* = 0.0305), and nitrofurantoin (*p* = 0.0002) resistance phenotype (Table 4). MDR-phenotype was equally distributed among community-acquired (64.5%) and hospital-acquired (61.3%) isolates. Moreover, MDR-phenotype was associated to male infections (83.33%, *p* = 0.02329). MDR-phenotype (69.6%) as well as the FQ-resistant phenotype (69.6%, p = 0.0008) was also prevalent among older adults (> 60 years old).

### Phylogenetic characterization, virulence genes, and their association to MDR-phenotype

All seven phylogenetic lineages (A, B1, B2, C, D, E and F) and cryptic clades were found on the 110 urinary *E*. *coli* isolates. The phylo-groups D (23.6%) and A (19.1%) were the most commonly found, followed by B1 (15.5%), C (13.6%), B2 (11.8%), F (10%), cryptic clades (5.5%) and phylo-group E (0.9%). The different phylo-groups and cryptic clades were distributed in both hospital and community settings, except the phylo-group E which was only distributed in the hospital-acquired infections. MDR strains were distributed into all phylogenetic-groups: 26% (16/70) to A, 17.1% (12/70) to D, 15.7% (11/70) to B1, 12.9% (9/70) to C, 11.4% (8/70) to B2 and F, and 6.7% (5/70) to cryptic clades. Phylo-group D was significantly associated to MDR-phenotype (*p* = 0.0339) as well as FQ-resistant phenotype (26%, *p* = 0.0173). Phylo-groups B2 and D were more common among community-acquired isolates (18.2, 9.1%) than hospital-acquired isolates (5.5, 2.7%), as well as in females (20.9, 7.3%) than in males (4.5, 2.7%) but not significantly associations were found.

Overall, *fimH* (71.8%), *fyuA* (68.2%), and *agn43* (54.5%) were the virulence genes with the highest distributions among isolates, while *afa/dra* (8.2%) and *cnf1* (2.7%) had the lowest. Others virulence genes tested had the following distribution: *chuA*, 49.1%; *papC*, 42.7%; *kpsMTII*, 37.3%; *vat*, 20%; *yfcV*, 20%; *sfaS* 10%, and *hlyA*, 9.1% (Table 5). Isolates from hospital-acquired infections presented slightly more prevalence of virulence genes than community-acquired infections (35.2% vs 30.2%). Prevalence of *fyuA* (71.0% vs 67.1%), *papC* (48.4% vs 40.5%), *yfcV* (25.8% vs 55.7%), *hlyA* (12.9% vs 7.6%), *cnf1* (3.2% vs 2.5%), *kpsMTII* (58.1% vs 29.1%) and *chuA* (51.6% vs 48.1%) were higher among UPEC isolated from hospital-acquired than community-acquired infections. However, only the *kpsMTII* was significantly associated with hospital-acquired isolates (58.1% vs 29.1%, *p* = 0.0047, Table 5). Comparing male vs female patients, isolates from females had a higher mean of virulence genes compared those isolates from male origin (21.5% vs 7.9%, respectively). Moreover, the *yfcV* was significantly more associated to male infections (41.7%, *p* = 0.0026). The presence of the *chuA* gene was associated to UPEC isolated from children (*p *= 0.0483). Furthermore, the presence of *vat* was associated to UPEC isolates from adults (*p *= 0.0153).

**Table 5:** Distribution of virulence genes among UPEC strains, n (%)

Virulence genes	Total isolates, *n* = 110 (%)	MDR, *n* = 70 (%)	Male, *n* = 24 (%)	Female, *n* = 86 (%)	*p	Hospital-acquired, *n* = 31 (%)	Community-acquired, *n* = 79 (%)	*p
*fyuA*	75 (68.2)	46 (65.7)	14 (58.3)	61 (70.9)		22 (71.0)	53 (67.1)	
*fimH*	79 (71.8)	51 (72.9)	20 (83.3)	59 (68.6)		21 (67.7)	58 (73.4)	
*sfaS*	11 (10)	6 (8.6)	2 (8.3)	9 (10.5)		2 (6.5)	9 (11.4)	
*afa/dra*	9 (8.2)	5 (7.1)	0 (0.0)	9 (10.5)		2 (6.5)	7 (8.9)	
*papC*	47 (42.7)	27 (38.6)	12 (50.0)	35 (40.7)		15 (48.4)	32 (40.5)	
*agn43*	60 (54.5)	39 (55.7)	14 (58.3)	46 (53.5)		16 (51.6)	44 (55.7)	
*yfcV*	22 (20)	15 (21.4)	10 (41.7)	12 (14.0)	0.0026	8 (25.8)	14 (17.7)	
*vat*	22 (20)	13 (18.3)	7 (29.2)	15 (17.4)		6 (19.4)	16 (20.3)	
*hlyA*	10 (9.1)	7 (10.0)	4 (16.7)	6 (7.0)		4 (12.9)	6 (7.6)	
*cnf1*	3 (2.7)	2 (2.9)	1 (4.2)	2 (2.3)		1 (3.2)	2 (2.5)	
*kpsMTII*	41 (37.3)	25 (35.7)	9 (37.5)	32 (37.2)		18 (58.1)	23 (29.1)	0.0047
*chuA*	54 (49.1)	32 (45.7)	11 (45.8)	43 (50.0)		16 (51.6)	38 (48.1)	

* Only statistical significant values are shown

Virulence genes *fimH* (72.9%), *fyuA* (65.7%), *agn43* (55.7%) and *chuA* (45.7%, Table 5), were highly distributed among MDR strains. However none virulence genes were significantly associated to MDR-phenotype or FQ-resistant isolates. Nevertheless, *fimH* (69.2%), *fyuA* (67.3%) and *chuA* (57.7%) were highly distributed among FQ-resistant bacteria.

### Identification of virulence, and antimicrobial resistance genes by microarray analysis

Microarray analysis was done on 18 *E. coli* isolates belonging to the phylo-groups B2, D and F. Overall, 145 virulence and 40 antimicrobial resistance genes among the 315 and 82 genes and variants investigated were detected at least once in one or more of the isolates. The total number of virulence genes per isolate ranged from 18 to 63 genes. The median number of the virulence genes per isolate was 35. Microarray hybridizations demonstrated that all the *E. coli* isolates tested possessed virulence genes related to a pathotype including ExPEC and diarrheagenic *E*. *coli* (DEC). The total number of antimicrobial resistance genes per isolate ranged from 0 to 21 genes with a median number of 6.5 genes. Since the microarray carries a large set of virulence factors, numerous incomplete ExPEC that would normally be missed in a PCR-based assay were found. Thus, various unusual gene combinations were discovered, such as ExPEC pathogenic profiles with assorted EPEC genes coding for the type III secretion system 2 proteins ErpJ and SpaS (gene *eprJ* and *epaS*, respectively). Moreover, detection of virulence (mean 39.0 ± 16.9) and antimicrobial resistance (mean 9.0 ± 1.9) genes were higher on hospital-acquired UTIs isolates compared to community-acquired infections (Table 6). Higher frequency of virulence genes was found among isolates retrieved from females compared to those from males (36.25 ± 17.04 mean vs 31.66 ± 13.00 mean, respectively), as well as those isolates from children vs adults (36.58 ± 7.4 mean vs 26.6 ± 16.39 mean, respectively). In addition, antimicrobial resistance genes were also detected on higher ratios among isolates retrieved from males compared to those from females (7.88 ± 5.53 mean vs 4.0 ± 3.03 mean, respectively); a similar situation was observed when strains isolates from children vs those from adults were compared (14.60 ± 5.79 mean vs 4.4 ± 2.60 mean, respectively). Interestingly, the maximum number of antimicrobial resistance genes was found among UPEC isolates from children.

**Table 6:** Virulence and antimicrobial resistance genes detected by microarray analysis exclusively among *E. coli* B2, D and F isolates from UTIs patients

	Type of infection	
	Hospital-acquired (*n *= 4)	Community-acquired (*n *= 14)
Virulence genes (mean)	39	32
Antimicrobial resistance genes (mean)	9	6
Phylo-groups	B2 (*n* = 2) and D (*n* = 2)	B2 (*n* = 9), D (*n* = 3) and F (*n* = 2)
Virulence genes function		
Resistance	*bla*_TEM_, *bla*_OXA-1_, *bla*_PSE-1_, *bla*_CTX-M-15_, *bla*_CMY-2_, *mphA*, *ant(3″)*-*Ia (aadA1)*, *aac(3)*-*IIa (aacC2)*, *tet(A)*, *tet(B)*, *tet(30)*, *tet(R), sulI sulII*, *qnrA*, *aac(6′)*-*Ib*-*cr*, class 1 integron	*bla*_TEM_, *bla*_OXA-1_, *bla*_OXA-9_, *bla*_PSE-1_, *bla*_CTX-M-15_, *bla*_CTX-M-12_, *bla*_OXY/K1_, *ant(3″)*-*Ia (aadA1)*, *aac(3)*-*IIa (aacC2)*, *aac(3)*-*IV*, *aph(3′)*-*Ia (aphA1)*, *aph(3′)*-*IIa (aphA2)*, *aph3 (strA)*, *dhfrI*, *dhfrVII*, *catI*, *sulI*, *sulII*, *tet(A)*, *tet(B)*, *tet(30)*, *mphA*, class 1 integron, class 2 integron
Adhesins	*afaD*, *afaD8*, *afaE1*, *afaE2*, *afaE5*, *bfpA*, *fimC*, *ibeB*, *csgA*, *csgE*, *f165(1)A*, *fimA*, *fimH*, *iha*, *lpfA*(*O113*), *papA(10)*, *papA(11)*, *papA(16)*, *papA(8)*, *papC*, *papGI2*, *fitA*, *sfaHII*, *sfaD*	*afaE1*, *afaE2*, *afaE5*, *cofA*, *lpfA2*, *fimC*, *csgA*, *csgE*, *csuA*, *F17b*-*A*, *F17d*-*A*, *facA*, *lpfA1*, *fimA*, *iha*, *lpfA(O113)*, *lpfA*, *papA(8)*, *papA(9)*, *papA(10)*, *papA(12)*, *papA(13)*, *papA(15)*, *papA(40)*, *papA(48)*, *papC*, *papGII*, *papGIV*, *fitA*, *sfaHII, sfaA*
Colcins and microsins	*cvaC*, *mchB*	*cda*, *ce1a*, *cib*, *cvaC*, *mcbA*.
Toxins	*Sat*	*astA*, *astA(2)*, *cdtB*-*4*, *cnf1*, *sat*
Iron acquisition or transport system	*chuA*, *fepC*, *fyuA*, *iroN*, *iroN(2)*, *irp1*, *irp2*, *iucD*, *iutA*, *iutA(2)*, *iutA(UPEC)*, *sitA*, *sitD*	*chuA*, *fepC*, *fimH*, *fyuA*, *iroN(2)*, *irp1*, *irp2*, *iucD*, *iutA*, *iutA(2)*, *iutA(UPEC)*, *sitA*, *sitD*
Capsular and somatic antigens	*kpsMTII*, *neuC*	*kpsMTII*, *neuA, neuC*
Locus of enterocyte effacement (LEE)	*eae(delta)*	*eae(lambda)*, *espG*, *esta1*
ETT2 elements	*eivG*, *eprJ*	*eivG*, *eprJ*, *epaS*
Haemolysins and hemagglutinins	*hlyA*, *hlyE*, *hra1*	*bmaE*, *hlyA*, *hlyE*, *hra1*.
Various functions	*agn43*, *capU*, *ccdB*, *deoK*, *fliC*, *gad*, *iss*, *iss(3)*, *malX*, *ompA*, *ompT*, *ompT(2)*, *senB*, *traT*	*agn43*, *capU*, *ccdB*, *ibeA*, *ibeB*, *deoK*, *eaf*, *fliC*, *fliC(H7)*, *gad*, *invX*, *iss*, *iss(3)*, *malX*, *ompA*, *ompT*, *ompT(2)*, *senB*, *traT*
Newly recognized or putative *E. coli* virulence genes	*artJ*, *b1121*, *b1432*, *gimB(orf1), mviM*, *mviN*, *shf*, *usp*, *virK*	*artJ*, *b1121*, *mviM*, *mviN*, *set*, *shf*, *usp*, *virK*

### Distribution of plasmid-mediated quinolone resistance genes, and ESBL genes

Overall, 22% of the isolates were positive for *qnrA* (25 isolates), 20.9% (23 isolates) to *qnrB*, 6.4% (7 isolates) to *qnrS1*, 0.9% (1 isolates) to *qnrC*, 4.5% (5 isolates) to *qnrD*, and 6.4% (7 isolates) to *aac(6′)*-*Ib*-*cr* (Table 7). None isolates were positive to *qepA* gene. Isolates were taken to sequencing to obtain information on the QRDRs of the *gyrA* and *parC* genes, respectively. The sequencing results for the QRDR of *gyrA* and *parC* are summarized in Table 8. Only one isolate tested had no mutations in the QRDRs of *gyrA* and *parC* since was sensitive to ciprofloxacin (CIP), levofloxacin (LEV), and norfloxacin (NOR). The double mutations in Ser-83 → Leu and Asp-87 → Tyr or Asp-87 → Asn substitution in *gyrA* were present in 92% of the strain tested. Ser-80 → Ile substitution in *parC* were found in 92% of the isolates tested, which also were resistant to both ciprofloxacin and levofloxacin. The substitution Thr-66 → Ser (1 strain, CIP-resistant, LEV-resistant), Thr-66 → Asn (1 strain, CIP-resistant, LEV-resistant), and Thr-66 → Tyr (2 strains, CIP-resistant, LEV-resistant) were also found in *parC*. Other substitutions in *parC* as Glu-84 → Val (2 strains, CIP-resistant, LEV-resistant) and Glu-84 → Ala were also detected. Interestingly, one strain (isolated UEc 76) which was resistant to ciprofloxacin, levofloxacin and norfloxacin, belonging to B2 phylo-group and blaCTX-M-containing, also carried the double mutations in Ser-83 → Leu and Asp-87 → Asn substitution in *gyrA* and the triple mutation in *parC* Ser-80 → Ile, Thr-66 → Tyr and Glu-84 → Val.

**Table 7:** Distributions of PMQR and blaCTX-M genes among UPEC, n (%)

Virulence genes	Total isolates, *n* = 110 (%)	MDR, *n* = 70 (%)	Male, *n* = 24 (%)	Female, *n* = 86 (%)	*p	Hospital-acquired, *n* = 31 (%)	Community-acquired, *n* = 79 (%)
*qnrA*	25 (22.7)	15 (21.4)	7 (29.2)	18 (20.9)		9 (29.0)	16 (20.3)
*qnrB*	23 (20.9)	13 (18.6)	6 (25.0)	17 (19.8)		10 (32.3)	13 (16.5)
*qnrS*	7 (6.4)	3 (4.3)	3 (12.5)	4 (4.7)		2 (6.5)	5 (6.3)
*qnrC*	1 (0.9)	0 (0.0)	0 (0.0)	1 (1.2)		0 (0.0)	1 (1.3)
*qnrD*	5 (4.5)	4 (5.7)	1 (4.2)	4 (4.7)		3 (9.7)	2 (2.5)
*aac(6′)*-*Ib*-*cr*	7 (6.4)	4 (5.7)	4 (16.6)	3 (3.5)	0.0194	2 (6.5)	5 (6.3)
*bla*_CTX-M_	14 (12.4)	11 (15.7)	5 (20.8)	9 (10.5)		4 (12.9)	10 (12.7)

* Only statistical significant values are shown

**Table 8:** Characterization of UTI isolates

Strain	Resistance profile	Phylo-group	PMQR genes	QRDR mutations		β-lactamase-resistance-genes	Other resistant genes	Integron
				**∆GyrA**	∆ParC			
UEc 11	AMP, SXT	B2		NA	NA	*bla*_TEM_	*ant(3″)*-*Ia (aadA1)*, *aph3 (strA)*, *dhfrI*, *sulII*, *tet(B)*	Class 2 integron
UEc 22	AMP, SAM, AMC, CFZ, SXT, CIP, LEV, NOR	B2		S83 → L D87 → N	S80 → I T66 → N	*bla*_TEM_	*tet(B)*	
UEc 23	AMP, SAM, CFZ, CTZ, CAZ, CRO, FEP, SXT, CIP, LEV	F	*aac(6′)*-*Ib*-*cr*	NA	NA		*aac(3)*-*IIa (aacC2)*	Class 1 integron
UEc 27	GEN, AMP, SAM, AMC, SXT, CIP, LEV	B2		S83 → L D87 → N	S80 → I E84 → V	*bla*_PSE-1_	*sulI*, *tet(A)*	
UEc 30	GEN, SAM, AMC, SXT	B2		NA	NA	*bla*_OXA-1_	*ant(3″)*-*Ia (aadA1)*, *tet(A)*, *tet(30)*, *tet*^*®*^*, sulI*	Class 1 integron
UEc 58	AMP, SXT, CIP, LEV	B2		S83 → L D87 → N G114 → S	S80 → I	*bla*_TEM_	*dhfrVII*, *sulII*, *tet(B)*, *aph3 (strA)*, *mphA*	
UEc 65	AMP, CFZ, CAZ, CRO, FEP, CIP, LEV	D		S83 → L D87 → N G114 → S	S80 → I	*bla*_CTX-M-15_ *bla*_TEM_	*aac(3)*-*IIa (aacC2)*, *dhfrVII*, *mphA*, *sulI*, *tet(B)*	Class 1 integron
UEc 69	AMP, SXT, NIT	B2	*qnrA*	NA	NA	*bla*_TEM_	*sulII*	
UEc 75	AMK, GEN, AMP, SAM, AMC, TZP, CFZ, CTX, CRO, FEPCIP, LEV, NOR	B2	*qnrA*	S83 → L D87 → N	S80 → I T66 → Y	*bla*_OXY/K1_, *bla*_TEM_	*aac(3)*-*IIa (aacC2)*, *aph(3′)*-*Ia (aphA1)*, *aph(3′)*-*IIa (aphA2)*, *aph3 (strA)*, *sulI*, *sulII*, *tet(A)*, *tet(B)*	
UEc 76	CFZ, CAZ, CRO, FEP, CIP, LEV	B2		S83 → L D87 → N	S80 → I T66 → Y E84 → V	*bla*_CTX-M-15_ *bla*_OXA-1_	*tet(A)*, *tet(30)*	
UEc 84	GEN, TOB, AMP, SAM, TZP, CFZ, CTX, CAZ, CRO, FEP, SXT, CIP, LEV	D	*aac(6′)*-*Ib*-*cr*	S83 → L D87 → N	S80 → I	*bla*_CTX-M-15_, *bla*_OXA-1_ *bla*_TEM_ *bla*_PSE-4_	*tet(30)*, *mphA*	
UEc 99	AMP, SXT	B2	*qnrA*	None	None	*bla*_CTX-M-12_, *bla*_OXA-9_, *bla*_TEM_	*aac(3)*-*IV*, *aac(3)*-*IIa (aacC2)*, *aph3 (strA)*, *catI*, *sulI*, *sulII*	Class 1 integron
UEc 102	AMP, CFZ, CAZ, CRO, FEP, SXT, CIP, LEV	B2		S83 → L D87 → N G114 → S	S80 → I	*bla*_TEM_	*aph(3′)*-*Ia (aphA1)*, *sulII*	
UEc 104	AMP, SXT	D	*qnrA, aac(6′)*-*Ib*-*cr*	NA	NA	*bla*_CMY-2_, *bla*_CTX-M-12_, *bla*_OXA-1_	*aac(3)*-*IIa (aacC2)*, *sulI*, *tet(B)*, *mphA*	Class 1 integron
UEc 107		F		NA	NA			
UEc 108	AMP	D	*qnrA*	NA	NA	*bla*_TEM_	*sulI*	Class 1 integron
UEc 109	AMP, SAM	D	*aac(6′)*-*Ib*-*cr*	NA	NA	*bla*_TEM_	*sulII*	
UEc 110	GEN, TOB, AMP, SAM, CTX, SXT, CIP, NIT	B2		NA	NA		*aac(3)*-*IIa (aacC2)*, *tet(B)*	Class 1 integron

*AMK* Amikacin, *GEN* gentamicin, *TOB* tobramycin, *NET* netilmicin, *AMP* ampicillin, *SAM* ampicillin–sulbactam, *AMC* amoxicillin–clavulanic acid, *TZP* piperacillin–tazobactam, *CFZ* cefazolin, *CTX* cefotaxime, *CAZ* ceftazidime, *CRO* ceftriaxone, *FEP* cefepime; *SXT* trimethoprim–sulfamethoxazole, *CIP* ciprofloxacin, *LEV* levofloxacin, *NOR* norfloxacin, *NIT* nitrofurantoin, *ETP* ertapenem, *IPM* imipenem, *PMQR* plasmid-mediated quinolone resistance genes, *QRDR* quinolone resistance-determining region, *NA* not analyzed

ESBL genes were investigated through microarray and PCR assay. A total of 27 isolates (24.5%) suspected as cephalosporinase producers showed positive PCRs for *bla*_CTX-M_ (12.7%/14 isolates), *bla*_TEM_ (18.2%/20 isoaltes), *bla*_PSE_ (2.7%/3 isoaltes), and *bla*_OXA_ (9.1%/10 isolates). Sequencing of the *bla*_CTX-M_ amplicons identified the presence of *bla*_CTX-M-15_ in 7.2% of the strains (8 isolates), *bla*_CTX-M-12_ in 4.5% (5 isolates), *bla*_CTX-M-3_ in 1.8% (2 isolates), and *bla*_CTX-M-14_ in 1.8% (2 isolates). Furthermore, *bla*_ROB-1,_ *bla*_SHV-37_, and *bla*_CMY-2_ genes, were identified in one isolate (0.9%) through the microarray assay.

Overall *bla*_CTX-M_ genes and PMQR genes as well as mutation in QRDR were found in both community- and hospital-acquired infections as well as in male and female genders. The PMQR *qnrA* (21.4%), and *qnrB* (18.6%), were highly distributed on MDR-phenotype (Table 7). Isolates from males had higher prevalence of *aac(6′)*-*Ib*-cr gene compare to female isolates (16.6% vs 3.5%, *p* = 0.0194). Quinolone-resistance isolates also presented higher prevalence to *bla*_CTX-M_ compared to quinolone-sensitive phenotype isolates (21% vs 5.2%, *p *= 0.012).

## Discussion

In accordance with global trends, our results reveal higher prevalence of urinary tract infections in female patients than in males [7, 34–36]. The emergence of high rates of antibiotic resistance and MDR-phenotype from urinary tract infections related bacteria becomes a public health concern worldwide. In this study, more than 70% of the isolates showed resistance to trimethoprim–sulfamethoxazole (TMP–SXT) that is recommended as a first choice for UTI treatment [36]. Previous studies reported similar results [20, 36]. In agreement with Bouchillon et al. [34] in this study, UPEC isolates presented more than 40% resistance to ciprofloxacin and levofloxacin antibiotics. Moreover, resistance rates to antimicrobial drugs were higher in isolates from male than in those from female patients [37]. Interestingly, fluoroquinolones are widely used for the treatment of UTIs in male patients [36, 38]. It is possible that male infections may be more difficult to eradicate because of the higher rates of antibiotic resistance observed in strains isolated from males, which may lead to recurrent infections. These observations were similar to previous papers [35, 36, 39]. Susceptibility analysis of isolates to antibiotics prior to treatment choice is recommended.

Multidrug-resistant strain prevalence (63.3%) was higher in this work than in other study from Mexico, which reported only 16.4% of MDR strains [21]. While work of Paniagua-Contreras et al. [20], showed 97% of MDR strains. It is well known that susceptibility patterns may vary in different geographical regions and can be change over time [36]. In addition, an unexpected result was found, as the percentage of resistance to the antibiotics tested and MDR-phenotype were similar in hospital and community-acquired infections, indicating an important reservoir of resistance in both settings [34]. This could be due to household use of antibiotics before 2010, which would were acquired without prescription then. Moreover, resistance to cephalosporin and nitrofurantoin was associated to hospital-acquired infections. Co-resistance with fluoroquinolones and TMP–SXT was also observed in both hospital and community.

As detected by Ochoa et al. [21], phylo-group D, an important phylo-group in pathogenic ExPEC was associated to MDR-phenotype. Additionally, FQ-resistant strains were correlated to phylo-group D strains. Strains from hospital-acquired infections exhibited a greater number of virulence genes than those from community. In addition, *E. coli* isolates from hospital-acquired UTIs are correlated to the presence of *kpsMTII* gene that has been associated to pyelonephritis, a more severe infection of the upper urinary tract [40]. In agreement with other studies [20, 41], our results showed an important frequency of *fyuA*-encoding yersiniabactin receptor and, *fimH* encoding type 1 fimbrial adhesion.

Detailed analysis of a strains subset using microarrays, revealed that some of them from both, hospital- and community-acquired infections carried virulence related genes of enteroaggregative and diffusely adherent *E*. *coli* (EAEC and DAEC), including *capU* (cap locus protein, hexosyltransferasa), *deoK* (deoxyribokinase), *shf* (putative virulence factor, plasmid pAA2, similar to *Shigella flexneri* Shf), *virK* (similar to *Shigella flexneri* virulence protein VirK, plasmid pAA2) and *astA* gene (EAEC heat-stable enterotoxin 1). This was also reported in previous works [42–45], which noticed that EAEC virulence related genes were among the most frequent markers of diarrheagenic *E*. *coli* reported in ExPEC strains. The *deoK* operon is frequently associated with strains isolated from infected urine and blood and is part of a large genetic island carrying genes contributing to the strain intrinsic virulence and/or adaptive properties [43]. These strains, by acquiring the pAA plasmid, could become a potential diarrheal agent [42].

Another finding was the presence of enteropathogenic *E*. *coli* (EPEC) related genes on five UPEC isolates. These genes included the *eae* (coding for intimin, a protein involved in attaching and effacing lesions), as well as virulence related genes such as *espG* (protein secreted by the type III secretion system and translocated into host epithelial cells), *eprJ* (*E*. *coli* type III secretion system 2 protein ErpJ), *epaS* (*E*. *coli* type III secretion system 2 protein EpaS), and *eivG* (*E*. *coli* type III secretion system 2 protein EivG) [3, 46]. T3SS genes are not common in UPEC isolates, however genes encoding components of T3SS have been found [47, 48]. These unusual gene combinations illustrate genome plasticity displayed by UPEC strains that may result in new hybrids pathotypes [45, 49, 50].

In this study, UPEC strains carrying PMQR genes associated to quinolones resistance including *qnrA*, *qnrB*, *qnrS*, *qnrC* and *qnrD* as well as *aac(6′)*-*Ib*-*cr* were detected. The most common PMQR-genes identified were *qnrA* and *qnrB*, which is in contrast to previous studies were *aac(6′)*-*Ib*-*cr* gene detection was more frequent on ExPEC isolates [19, 51]. Moreover, *qnrA* and *qnrB* were frequently found as a part of MDR-phenotype. *qnrD* gene was found in 4.5% of the isolates. Previous papers reported *qnrD* in bacteria isolated from rooks [51], pigs [15] and humans clinical isolates of *Salmonella enterica* serovar Kentucky, serovar Bovismorbificans, *Proteus mirabilis* and *Morganella morganii* [52, 53], as well as in *E. coli* from dogs [54]. In accordance to others studies [19, 55, 56], *qepA* was not identified. Although PMQR genes provide a low level of FQ-resistance, they have been reported to favor the selection of additional chromosome-encoded resistance mechanism [57]. In our study, QRDR mutations in *gyrA* and *parC* were prevalent in community-acquired infections (89% vs 11%). Overall, mutations in QRDR were equally present among isolates from males and females patients.

In our study, ESBL genes *bla*_CTX-M_ (*bla*_CTX-M-15_, *bla*_CTX-M-14_, *bla*_CTX-M-12_, and *bla*_CTX-M-3_), *bla*_TEM_, *bla*_PSE_, and *bla*_OXA_ were detected. For the isolates carrying resistance genes, ESBL genes such as TEM, SHV and CTX-M, are the most widespread and frequently detected in *E. coli* [9]. In addition, CTX-M-15 is quite common in Mexico [58, 59].

## Conclusions

Our study describes significant antimicrobial resistance in *E*. *coli* UTI isolates from hospital and community in Mexico. Diverse genotypes and phenotypes including multidrug-resistance, fluoroquinolone resistance and carriage of virulence genes related to several enteropathogenic *E. coli* were found among UPEC isolates. Thus, continuous surveillance for antimicrobial resistance of UPEC is needed in order to prevent treatment failure and to improve the strategies to mitigate the occurrence of antibiotic resistance organisms and ensure the best treatment to UTI patients. This study highlights the importance of antimicrobial resistance of virulent *E. coli* from urinary tract infection in Mexico.

## Abbreviations

UTIs: urinary tract infections
MDR: multiple drug resistance
ExPEC: extra-intestinal pathogenic *E. coli*
ESBL: extended-spectrum beta-lactamase
QRQR: quinolone resistance-determining region
FQ-phenotype: fluoroquinolone resistance phenotype
